# Cancer cells grown in 3D under fluid flow exhibit an aggressive phenotype and reduced responsiveness to the anti-cancer treatment doxorubicin

**DOI:** 10.1038/s41598-020-68999-9

**Published:** 2020-07-21

**Authors:** Tayebeh Azimi, Marilena Loizidou, Miriam V. Dwek

**Affiliations:** 1 School of Life Sciences, University of Westminster, 115 New Cavendish St, London, W1W 6UW UK; 2 Division of Surgery and Interventional Science, Department of Surgical Biotechnology, UCL Medical School Royal Free Campus, Rowland Hill Street, London, NW3 2PF UK

**Keywords:** Breast cancer, Cancer microenvironment, Cancer models, Biomaterials - proteins, Experimental models of disease

## Abstract

3D laboratory models of cancer are designed to recapitulate the biochemical and biophysical characteristics of the tumour microenvironment and aim to enable studies of cancer, and new therapeutic modalities, in a physiologically-relevant manner. We have developed an in vitro 3D model comprising a central high-density mass of breast cancer cells surrounded by collagen type-1 and we incorporated fluid flow and pressure. We noted significant changes in cancer cell behaviour using this system. MDA-MB231 and SKBR3 breast cancer cells grown in 3D downregulated the proliferative marker Ki67 (P < 0.05) and exhibited decreased response to the chemotherapeutic agent doxorubicin (DOX) (P < 0.01). Mesenchymal markers snail and MMP14 were upregulated in cancer cells maintained in 3D (P < 0.001), cadherin-11 was downregulated (P < 0.001) and HER2 increased (P < 0.05). Cells maintained in 3D under fluid flow exhibited a further reduction in response to DOX (P < 0.05); HER2 and Ki67 levels were also attenuated. Fluid flow and pressure was associated with reduced cell viability and decreased expression levels of vimentin. In summary, aggressive cancer cell behaviour and reduced drug responsiveness was observed when breast cancer cells were maintained in 3D under fluid flow and pressure. These observations are relevant for future developments of 3D in vitro cancer models and organ-on-a-chip initiatives.

## Introduction

The importance of the tumour microenvironment (TME) as a modulator of cancer cell behaviour is well recognised and has led to the development of 3D in vitro models of cancer. This field has been driven by: (i) the need for laboratory models to enable interrogation of genetic, molecular and cellular events in real-time and (ii) the need for platforms to allow drug responses to be tested in a manner relevant to the clinic.

The TME comprises extracellular matrix (ECM) proteins and resident and infiltrating cells (for example fibroblasts, adipose, endothelial and immune cells) with physical features including capillary fluid flow and interstitial fluid pressure (IFP). The ECM, the major non-cellular component of the TME, comprises a complex mixture of macromolecules for example proteins, glycoproteins, proteoglycans and polysaccharides^1^. By supporting and organising cellular structures, the ECM contributes to major cellular processes such as cell proliferation, survival, migration and differentiation^2^ and interactions between cancer cells and the ECM results in alterations in response to anti-cancer treatments^3–5^. The biophysical properties of the TME includes ECM stiffness, fluid shear stresses and IFP. These important characteristics influence tumour cell invasion and metastasis^6–8^ and been shown to promote the migration of breast cancer cells grown in vitro in 3D^9^. Elevated IFP has been recognised as a tumour-associated phenomenon for several decades^10–12^, associated with the resistance of cancer cells to treatment, expression of mesenchymal markers and the collective invasion of cancer cells^13–16^. Recently microfluidic devices designed to enable the interplay between fluid flow and pressure and tumour cell behaviour have emerged^7^.

Triple-negative and HER2 positive breast cancers have the greatest rate of recurrence. In this study MDA-MB231 cells, a triple negative breast cancer (TNBC) subtype, and SKBR3, a HER2 enriched breast cancer subtype, were used to study the effect of 3D cell culture on responsiveness to doxorubicin an anthracycline chemotherapeutic agent which functions by intercalating DNA, inhibiting topoisomerase II and preventing DNA recombination. The aim was to evaluate, using a 3D model of breast cancer, the effect of increased fluid flow and IFP on cell proliferation, migration and sensitivity to doxorubicin.

## Results

**Figure 1:**
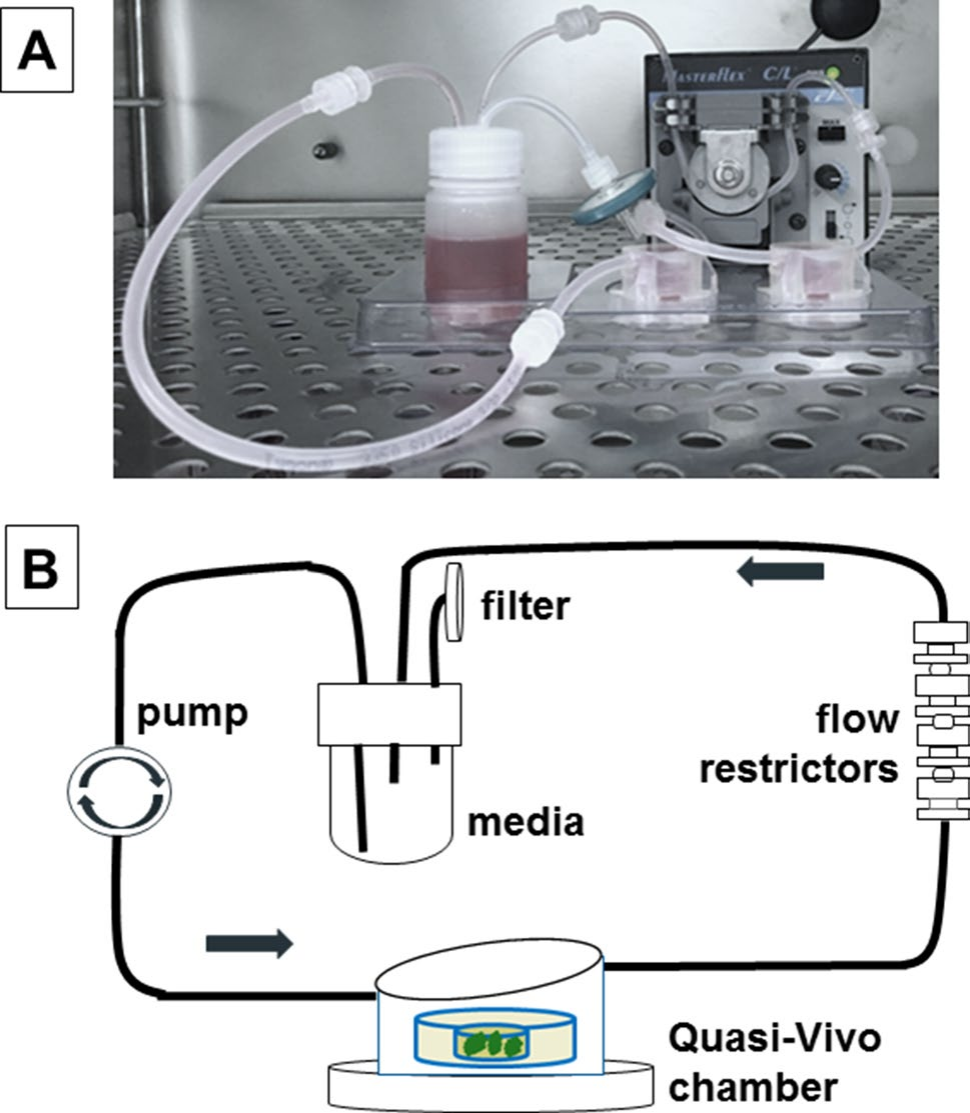
3D tumouroid flow system. (**A**) The experimental set-up housed in a laminar flow cabinet with Quasi-Vivo media reservoir, flow chamber and peristaltic pump. (**B**) Schematic view of the Quasi-Vivo fluid flow system with tumouroid housed inside the flow chamber, the peristaltic pump, flow restrictors, media reservoir, filter (0.22 µm) and direction of flow indicated.

Cancer cells were prepared as a central dense cancer mass and nested in collagen type-1. This tumouroid was then housed in a Quasi-Vivo flow chamber with in-line Luer-lock valves designed to increase the IFP (Fig. 1).

### Cell morphology and growth when cultured in 2D and 3D

**Figure 2:**
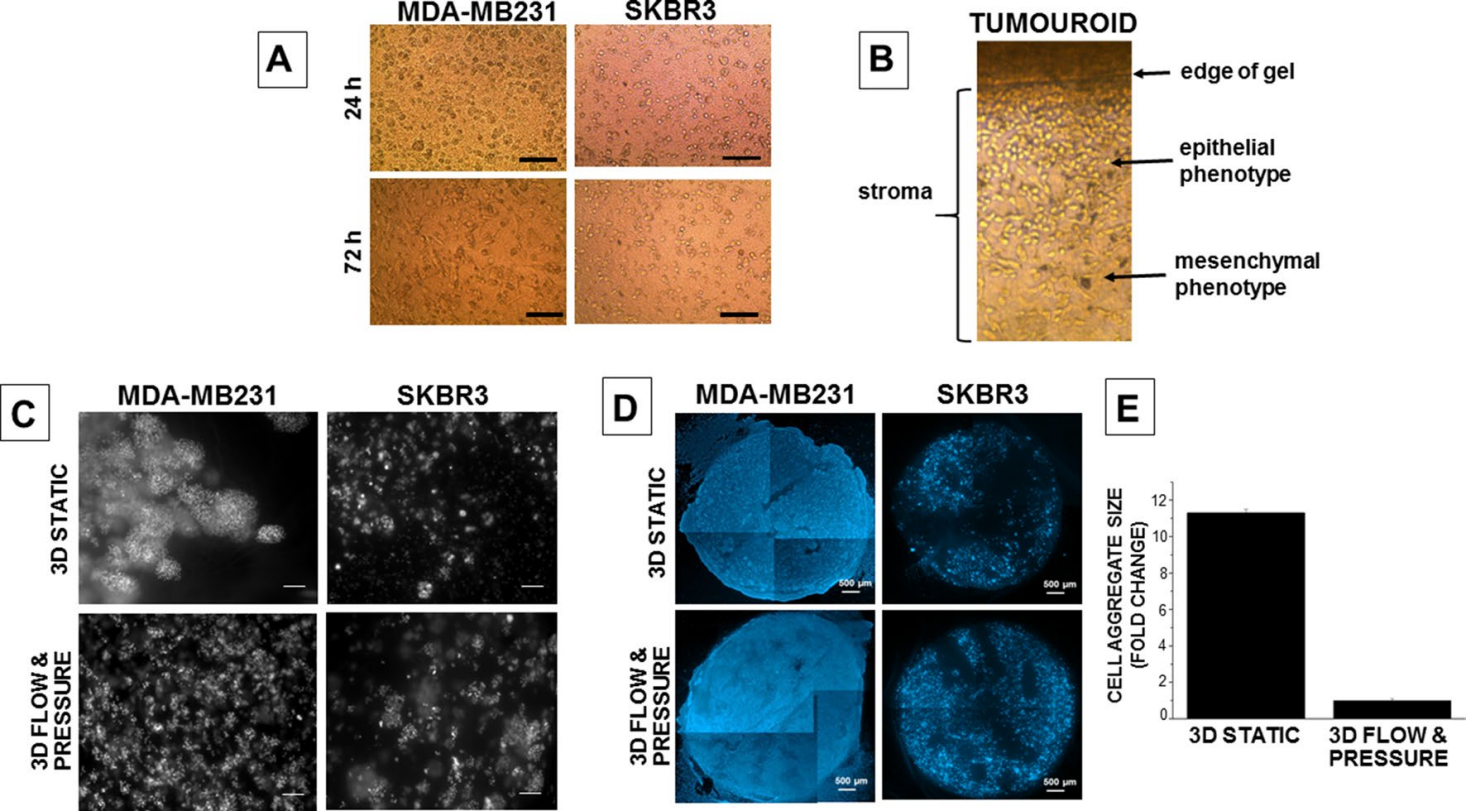
Cancer cells grown in 2D, 3D and 3D flow and pressure conditions. (**A**) MDA-MB231 and SKBR3 tumouroids prepared in 3D collagen type-1 and visualised using an inverted light microscope 24 and 72 h after tumouroid formation. (**B**) The invasion of MDA-MB231 cells into the surrounding stroma in the tumouroid. (**C**) Cellular aggregates in tumouroids maintained under static and flow and pressure conditions for 7 days (SKBR3) and 14 days (MDA-MB231). (**D**) Montage created using tumouroids imaged with a ×2.5 objective of an Apo Tom 0.2 microscope. (**E**) Fold change difference in size of MDA-MB231 cell aggregates formed in tumouroids under static or flow and pressure conditions. Scale bars 100 µm unless otherwise stated.

When the cancer cells were grown as tumouroids in collagen by day 7 they started to aggregate and form clusters, Fig. 2. A switch to an epithelial phenotype was initially observed for the MDA-MB231 cells at day 1 but by day 3 these cells had regained their characteristic mixed mesenchymal/epithelial phenotype both within the centre of the cancer mass and at the invasive edge abutting the acellular stroma. SKBR3 cells retained their epithelial morphology in 3D throughout. When MDA-MB231 cells were grown as tumouroids and exposed to flow and pressure they exhibited 11-fold smaller cellular aggregates compared with cells maintained as static tumouroids, Fig. 2. The cellular aggregates formed by the SKBR3 cells were, however, of a similar size, irrespective of the 3D culture conditions.

**Figure 3:**
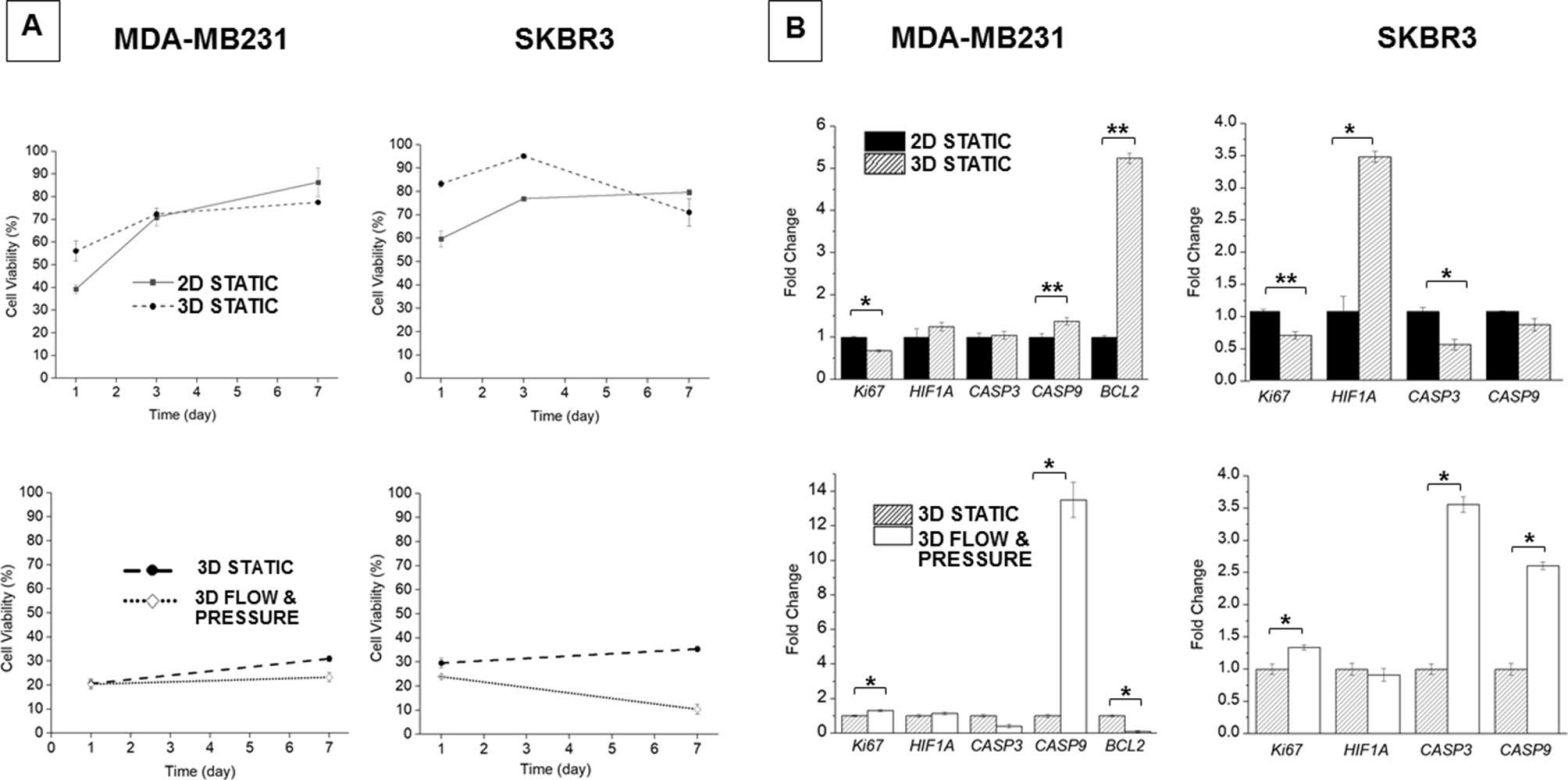
Cell viability and expression-levels for genes associated with apoptosis, proliferation and hypoxia for breast cancer cells maintained under different culture conditions. Upper panels: 2D monolayer culture and 3D tumouroid. Lower panels: 3D tumouroids in static culture and under flow and pressure conditions. (**A**) Reduction of Alamar blue reagent, indicating higher metabolic activity/cell viability over time. (**B**) Expression-levels of genes associated with apoptosis, hypoxia and proliferation in MDA-MB231 and SKBR3 cells. R = 2^−ΔΔCT^, i.e. fold change relative to 2D (upper panel) and static (lower panel). Mean average values ± SD (n = 3). Asterisks denote significant differences in gene expression, paired Student’s t-test; *P < 0.05; **P < 0.01; ***P < 0.001.

The growth rate of MDA-MB231 and SKBR3 cells cultured as 3D tumouroids was compared with 2D monolayer cultures using Alamar Blue reagent, Fig. 3. Fewer cells were seeded for the 2D culture (20,000 cells/well) compared to 3D culture (50,000 cells/tumouroid) due to limited space for attachment of cells in the 2D cultures. MDA-MB231 cells exhibited a reduced growth rate when cultured as tumouroids compared to 2D cell culture, and in flow and pressure conditions compared to static cell culture. The growth rate for SKBR3 cells propagated in 2D and tumouroids was similar until day 3, thereafter the growth rate of SKBR3 significantly reduced in the tumouroids. The same trend was observed when SKBR3 cells were grown as tumouroids under flow and pressure compared to static conditions.

### Proliferation, hypoxia, EMT, HER2 and apoptosis-related gene expression

To investigate the molecular mechanism(s) underlying the decreased growth rates in the tumouroids the expression levels of genes for proliferation, hypoxia and apoptosis was assessed, Fig. 3. Comparing the 3D tumouroids with the 2D cultures, the proliferation marker Ki67 was significantly downregulated and HIF1A was significantly upregulated in SKBR3 cells. Variable expression levels for caspase-3, caspase-9 and Bcl2 were observed when cells were grown in 3D under flow and pressure. SKBR3 grown under 3D static conditions exhibited CASP3 levels that were not consistent with CASP9, this may reflect the inherent heterogeneity of the multi-clonal SKBR3 cancer cell line. Ki67 levels increased significantly when tumouroids were maintained under flow and pressure compared to static conditions; HIF1A levels remained unchanged; pro-apoptotic markers, caspase-3 and caspase-9 were upregulated whilst the anti-apoptotic marker Bcl2 was significantly downregulated.

**Figure 4:**
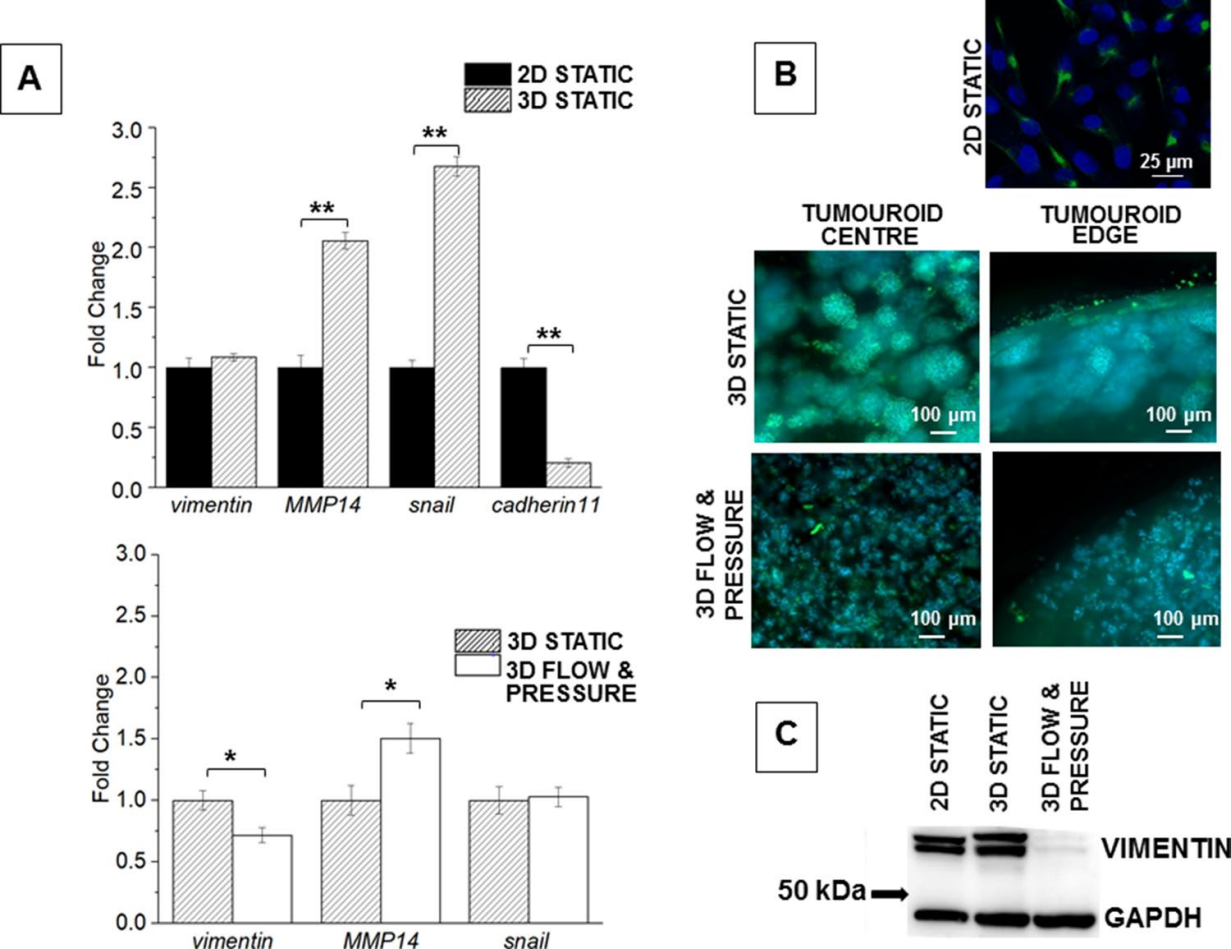
mRNA expression-levels for mesenchymal markers, immunofluorescent microscopy and western blot for vimentin in MDA-MB231 cells grown under different culture conditions. (**A**) Expression-levels of genes in MDA-MB231 cells. Upper panel: 2D monolayer culture and 3D tumouroid. Lower panel: 3D tumouroids in static culture and under flow and pressure conditions. R = 2^−ΔΔCT^ calculated by subtracting ΔCT 2D culture (control) from ΔCT 3D collagen; and by subtracting ΔCT 3D static from ΔCT 3D flow and pressure. Mean average values ± SD (n = 3). Asterisks denote significant differences in gene expression, paired Student’s t-test; *P < 0.05; **P < 0.01; ***P < 0.001. (**B**) Immuno-fluorescent microscopy of MDA-MB231 cells stained with anti-human vimentin (green). Upper panel: confocal microscopy of 2D monolayers, nuclei were counterstained with To-pro-3 (blue). Lower panels: fluorescence microscopy of cells in the centre and at the edge of the tumouroids after 14 days culture, nuclei were counterstained with DAPI. (**C**) Western blotting analysis of vimentin protein of MDA-MB231 cells grown under conditions as indicated. GAPDH was used as loading control.

Compared with 2D cell culture, tumouroids significantly upregulated both MMP14 and snail. Conversely, cadherin-11 expression levels decreased significantly and vimentin was unchanged, Fig. 4. When the tumouroids were grown under flow and pressure MMP14 was significantly upregulated whilst vimentin expression levels decreased significantly, and snail remained unaffected. Western blot analysis confirmed the results obtained with the qRT-PCR: there was no change in vimentin protein levels when MDA-MB231 cells were grown in 3D compared to 2D but a significant reduction in vimentin protein levels when tumouroids were maintained under flow and pressure. The antibody used recognises both the native and the phosphorylated forms of vimentin and the two bands observed on the western blots are consistent with the two isoforms of this protein, Fig. 4. Immunofluorescent microscopy confirmed the qRT-PCR and western blot results: cells grown in 2D and 3D had similar vimentin levels but this was reduced when cells were grown as tumouroids under flow and pressure.

**Figure 5:**
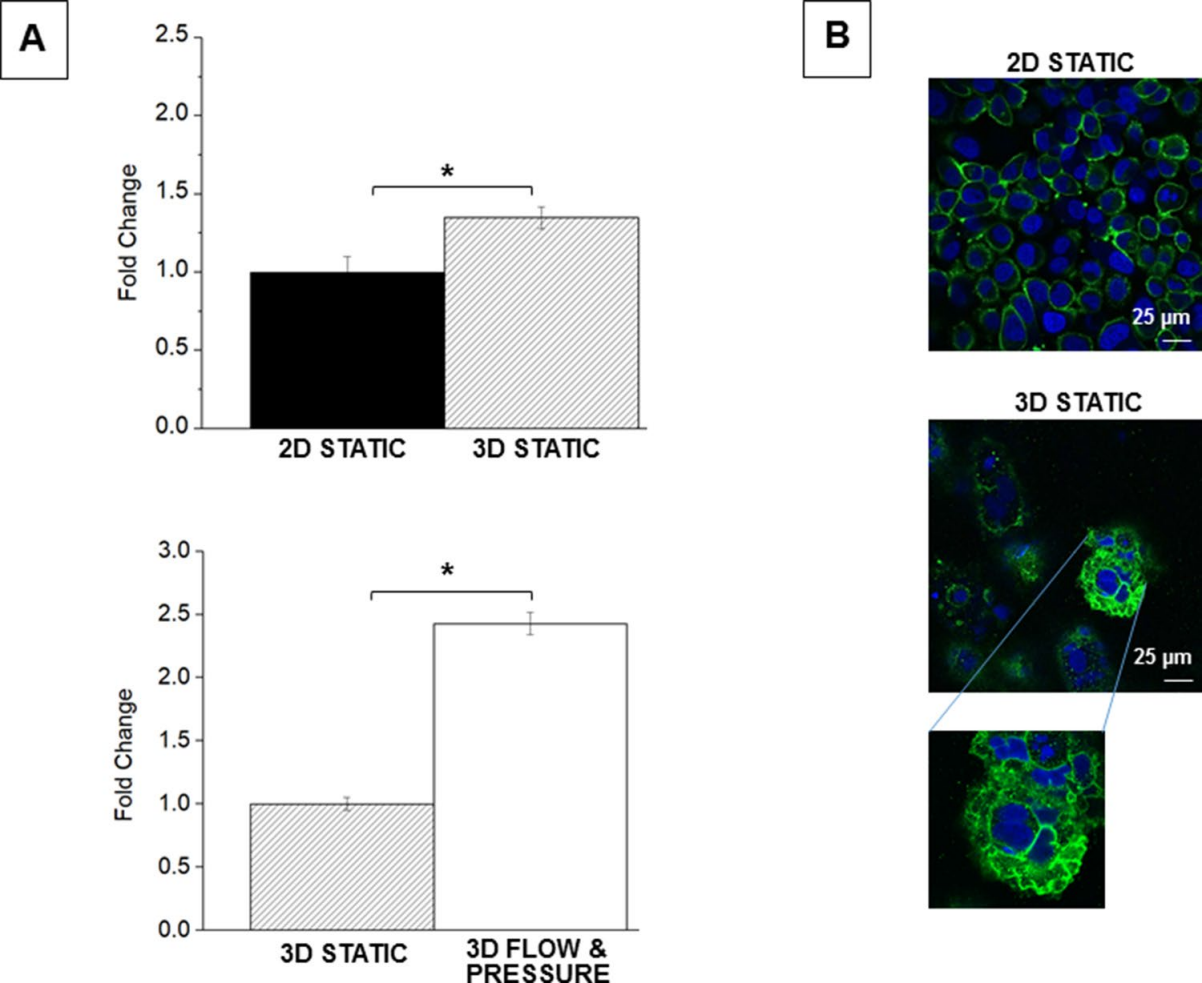
HER2 levels in SKBR3 cells grown under different culture conditions. (**A**) Expression-level of HER2. Upper panel: 2D monolayer culture and 3D tumouroid. Lower panel: 3D tumouroids in static culture and under flow and pressure conditions. R = 2^−ΔΔCT^ calculated by subtracting ΔCT 2D culture (control) from ΔCT 3D collagen; and by subtracting ΔCT 3D static from ΔCT 3D flow and pressure. Mean average values ± SD (n = 3). Asterisks denote significant differences in gene expression, paired Student’s t-test; *P < 0.05; **P < 0.01; ***P < 0.001. (**B**) Immuno-fluorescent microscopy of SKBR3 cells stained with anti-human HER2 (green). Upper panel: confocal microscopy of 2D monolayers, nuclei were counterstained with To-pro-3 (blue). Lower panel: fluorescence microscopy of HER2 levels within cells maintained as 3D tumouroids.

HER2 is the target for therapy with monoclonal antibodies such as trastuzumab and pertuzumab. SKBR3 cells are enriched in HER2 and are often used in studies concerned with the cellular behaviour and drug responsiveness of this breast cancer subtype. HER2 was upregulated when cells were grown in 3D and further significantly increased when tumouroids were maintained under flow and pressure, Fig. 5.

### Sensitivity to the anti-cancer agent doxorubicin (DOX)

**Figure 6:**
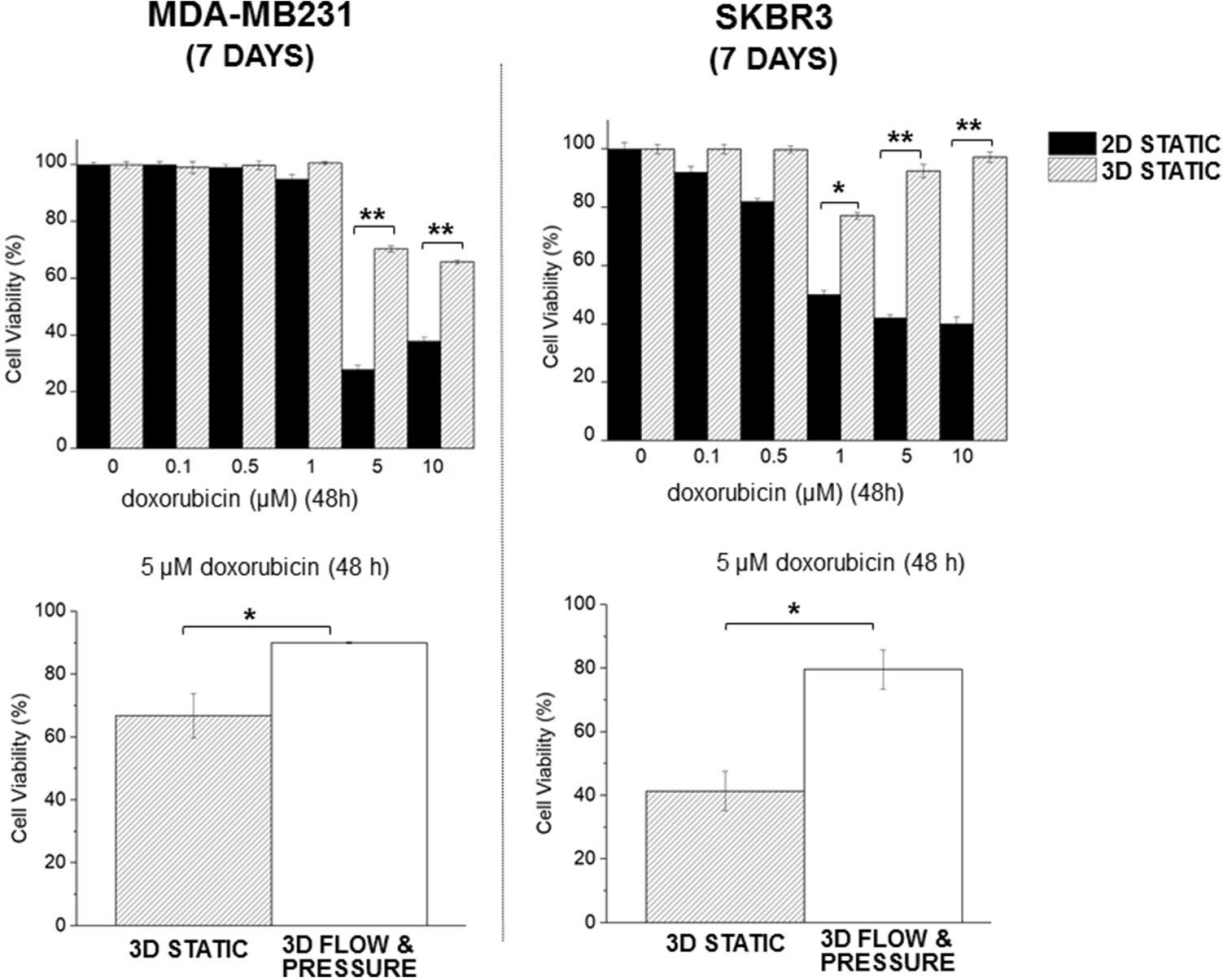
Sensitivity to doxorubicin of MDA-MB231 and SKBR3 cells were grown as monolayers and as tumouroids for 7 days. Cells were exposed to doxorubicin, at the concentrations shown, for 48 h. The cellular viability was measured using the Alamar blue assay. Mean average values ± SD (n = 3) are shown. Asterisks denote significant differences in cell viability, paired Student’s t-test; *P < 0.05; **P < 0.01; ***P < 0.001.

MDA-MB231 and SKBR3 cells were treated with DOX and cell growth assessed using the Alamar Blue assay, Fig. 6. When grown as 2D monolayers MDA-MB231 cells showed a significant decrease in viability following exposure to DOX (5 µM and 10 µM) and when grown in 3D were less sensitive to DOX. SKBR3 cells showed a significant decrease in viability when exposed to concentrations of ≥ 0.5 µM DOX in 2D and concentrations of ≥ 1 µM in 3D. Overall, both cell types showed reduced sensitivity to DOX when grown as 3D tumouroids compared to 2D culture. The application of flow and pressure to tumouroids further significantly reduced the cancer cell sensitivity to DOX.

## Discussion

In this study the growth and behaviour of breast cancer cells and sensitivity to DOX was investigated when cells were grown in 2D and 3D and under flow and pressure. Maintaining the cancer cells in 3D and in flow conditions resulted in a reduction in cell growth rates and also a reduction in the cancer cell sensitivity to DOX.

Collagen type-I has been widely used as a scaffold for 3D cell culture due to its physiological abundance^17^. Most reports of 3D cancer cell culture experiments describe loose cancer cell aggregates with a high liquid-to-cell ratio. Such models do not reflect the cell density or the stiffness of the ECM observed in vivo for solid tumours. In this study the RAFT system (Lonza) was used, this utilises absorbers to remove excess liquid from the collagen hydrogel resulting in a 40-fold increase collagen density. These 3D tumouroids therefore incorporate the mechanical forces of ~ 20 kPa found in tumours and exhibit oxygen levels equivalent to pathological hypoxia^17^, this has led to these tumouroids being described as ‘physiological tissue mimics’ (Supplementary Figure S1).

To our knowledge, this is the first report in which 3D cancer cultures of defined stiffness have been maintained under flow and pressure conditions. MDA-MB231 and SKBR3 cells in 3D exhibited significantly lower rates of proliferation than 2D monolayer cultures. This observation is consistent with other studies, for example, HT29 colorectal cancer cells prepared in our laboratories using the same RAFT system^18^. Similarly, it has been reported that U87, U251 and HS683 glioma cells cultured in 3D in collagen showed reduced growth rates over a 10 day period compared to 2D culture^19^, this may reflect limited diffusion of nutrients from the media into the centre of the 3D constructs.

Epithelial-mesenchymal transition (EMT) is characterised by increased levels of snail, MMP14, cadherin-11 and vimentin and is associated with cancer invasion and metastasis. EMT is induced as a result of the mechanical properties of the ECM^19–21^. HT29 and HCT116 colorectal cancer cells grown as tumouroids in 3D culture in our laboratory have previously been shown to exhibit increased levels of vimentin^22^. In this study, vimentin levels were unchanged when MDA-MB231 cells were maintained as tumouroids and the protein levels were significantly decreased when cells were grown under flow and pressure. This finding does not concur with a report by Piotrowski-Daspit et al. where increased expression levels of vimentin in MDA-MB231 cells (cultured in 3D in collagen subjected to interstitial hypotension applied through hydrostatic fluid pressure) was observed^23^. More work is required to understand the interplay between the TME and EMT when cancer cells are grown under fluid flow and pressure. The increased levels of phosphorylated vimentin in MDA-MB231 cells maintained in 3D suggests signal pathway activation.

Cancer cell invasion requires physical space within the ECM and the degradation of the ECM by enzymes such as the zinc dependent matrix metalloproteinases (MMPs) provides such space. Membrane bound MMP-14, for example, cleaves collagen type-I and other ECM components^24^. MMP-14 expression levels was significantly upregulated when MDA-MB231 cells were grown in 3D under flow and pressure. A similar observation has been reported when mammary fibroblasts were grown in 3D (non-compressed) collagen scaffolds compared to 2D monolayer culture^25^ and when the normal breast epithelial cell line MCF-10A was grown in 3D on collagen type-1^26^. A 3D model of ovarian cancer in which MMP14 was upregulated exhibited clustered β1-integrin at the cell surface^27^, subsequent knock-down of β1-integrin in MCF-7 and MDA-MB231 breast cancer cells grown in 3D resulted in down-regulation of MMP14^28^. This may represent a mechanism for induction of MMP-14 for the MDA-MB231 cells grown under flow and pressure described here.

Cadherin switching is also a feature of mesenchymal transition, it occurs through the action of snail and activation of associated cell signalling pathways^29,30^. When snail was knocked down in MDA-MB231 reduced cellular invasion in vitro and decreased lymph node metastases in vivo was reported^31^. 3D models of other epithelial cancers cultured in collagen have shown increased expression levels of snail^32,33^ mediated by TGF-β and downstream effector molecules. In the tumouroid model reported here snail expression levels were significantly increased when MDA-MB231 cells were grown in compressed collagen but remained unchanged under flow and pressure conditions. To date there have been limited reports on the effect of flow and pressure on mesenchymal markers in 3D models of cancer. Applying sheer stresses (1 dyne/cm^2^) via a 3D cell culture microfluidic device resulted in upregulation of α-SMA and snail and downregulation of VE-cadherin in human umbilical cord endothelial cells^34^. Snail expression levels were similarly increased in MDA-MB231 cells grown as collagen scaffolds and under hypotensive conditions compared to static and other pressure profiles^23^. In our study, the mRNA level for snail was increased when cells were maintained as 3D tumouroids but remained unaltered under flow and pressure conditions—this may reflect differences in the density of the collagen scaffold and flow pressure parameters applied in the different studies.

A significant reduction in cadherin-11 expression levels was observed when MDA-MB231 cells were grown as 3D tumouroids whilst cells grown as spheroids in ultra-low attachment plates showed significant upregulation of cadherin-11 (data not shown). These findings are at odds with reports that invasive breast tumours^35^ and cancer cells (MDA-MB231) metastatic to bone exhibit elevated levels of cadherin-11^36^.

Given the important role of 3D model systems as platform technologies in the oncology drug discovery pipeline it is imperative that the effect of culture conditions on cell membrane receptor levels are assessed, particularly as these are frequently the target for anti-cancer therapies. We observed an increase in HER2 levels when SKBR3 cells were grown in 3D and this was further attenuated under flow and pressure. Similarly, in a study using poly-HEMA coated plates, with an absence of ECM components, breast cancer cells BT474 and HCC1954 exhibited elevated HER2 protein levels^37^ and overexpression of HER2 was reported when the normal breast cell line MCF10A was maintained in agarose/Matrigel^38^. We observed spheroid-like aggregates when SKBR3 cells were grown as tumouroids and a significant increase in HER2 expression was observed when SKBR3 cells were grown as spheroids in ultra-low attachment plates without collagen (data not shown). These results indicate that the cell–cell interactions in 3D modulate HER2 levels and that increased HER2 levels do not arise from the interaction between cancer cells and the ECM alone.

3D cellular scaffolds, organoids and organ-on-a-chip models offer potential for drug discovery in oncology but the majority of in vitro 3D cancer models are usually free-floating or are made with soft hydrogels. Such systems do not mimic the physical properties of tumours^39^. In this study we observed a significant decrease in the responsiveness of breast cancer cells to DOX treatment when cells were cultured as dense 3D tumouroids, compared to 2D monolayers, was well as when cells were grown under flow and pressure compared to static conditions. This reduced responsiveness may be due to decreased diffusion of drug through the dense matrix and through the cell aggregates^40^, increased levels of HIF1A—indicative of hypoxic conditions within the tumouroid^41,42^ and anti-apoptotic signals through integrin-mediated cell–matrix adhesion^43^. Consistent with the results presented here, when the human ovarian cancer cell line OV-MZ-6 was grown as 3D spheroids a 50% increase in cell viability was observed (compared to monolayers) following treatment with paclitaxel^3^. Resistance to cytotoxic drugs for example cisplatin or mitoxantrone has been reported in MCF-7 and MDA-MB231 cells grown in type-1 collagen and β1-integrins play a key role in drug resistance^44^. A recent report described increased resistance to the chemotherapeutic agents Ara-C and DNR of acute lymphoblastic leukemia (Jurkat) cells grown on scaffolds coated with type-I collagen and this was associated with the overexpression of discoidin domain receptor 5. When the cervical cancer cell line HeLa was maintained as spheroids a decreased sensitivity to DOX was observed compared to cells grown in 2D^45^. MCF7 and MDA-MB231 breast cancer cells grown in PEG-fibrinogen hydrogels, housed in a microfluidic chip exhibited a decrease in response to paclitaxel and DOX^46^.

In this report we provide a detailed understanding of breast cancer cell behaviour when the cells were maintained as 3D tumouroids under fluid flow. Recently other fluidic systems have become available for example PhysioMimix (CN-Bio, UK); Mimitas (Leiden, Netherlands); these ‘off-the-shelf’ approaches augment bespoke systems developed in individual laboratories^47^, alongside this the emergence of ‘tumour-on-a-chip’ technology in which fluid flow is integral continues apace^48^. In these approaches it is important to ensure that the flow rates and shear forces delivered to the cancer cells are benchmarked against in vivo parameters. Concordance studies with different 3D cancer model systems under flow are now an important aim, the realisation of this will enable standardisation and adoption into the pharmaceutical drug development pipeline and is expected will result in reductions in the use of in vivo model systems^49–51^.

This report is the first to describe a reproducible off-the-shelf approach in which cancer cells were prepared as a dense 3D mass surrounded by an acellular ECM, creating a tumouroid construct maintained under fluid flow and pressure. Exposing the tumouroids to physiological conditions of flow and pressure resulted in changes in their growth, morphology and sensitivity to chemotherapeutic challenge. This model system provides key evidence of the role of tissue density and fluid flow and is relevant to researchers using 3D models as cancer drug testing platforms.

## Methods

All materials were supplied by Sigma-Aldrich, Poole, Dorset, UK unless otherwise stated.

### Cell culture

MDA-MB231 (triple negative) and SKBR3 (HER2 positive) human breast adenocarcinoma cells were obtained from the ATCC. Cells were maintained in high glucose Dulbecco’s modified Eagle’s medium (DMEM) supplemented with 10% v/v heat inactivated foetal bovine serum (FBS). 3D constructs “tumouroids”, comprising a central cancer mass embedded in a stromal compartment were manufactured using the RAFT 3D culture system (Lonza, UK) as described previously^22^. Briefly, collagen type-I hydrogel was prepared by addition of 2.8 mL of minimal essential medium (10X MEM) to 22.4 mL rat-tail collagen type-I, 2 mg/mL (First Link, UK) and 1.624 mL neutralising solution (1.6 M NaOH in 840 mM HEPES buffer). In order to seed 50,000 cells in each well, 48 × 10^5^ cells in 1.2 mL volume were added to the solution and mixed gently. 240 µL of the combined collagen-cell mixture was transferred to each well of a 96 well plate and incubated for 15 min at 37 °C allowing the hydrogel to set. RAFT absorbers were applied for 15 min to remove excess liquid thereby increasing the density of the collagen matrix. To create complex tumouroids, cancer masses were transferred into the centre of each well of a 24 well plate. Acellular stroma was added around the cancer mass and liquid was removed using RAFT absorbers, and the final product (100 µm thickness) was covered with media (DMEM plus FBS). This configuration enabled tumouroids to fit snugly inside flow chambers.

### Fluid flow and pressure

To generate the flow environment the Quasi-Vivo system (Kirkstall, UK) was used. Tumouroids were nested in QV-500 polydimethylsiloxane chambers (biocompatible, transparent, flexible, gas permeable silicone) and connected via inlet and outlet tubes in a ‘series’ configuration. A peristaltic pump was used to generate a pulsatile laminar unidirectional flow of media at various flow rates. A flow rate of 550 µL/min, corresponding to an IFP of 0–3 mmHg consistent with normal breast tissue IFP^52^ was used; whilst 19 mmHg, consistent with breast tumour IFP was achieved by the addition of four one-way Luer check-valves, biocompatible, female-male styrene acrylonitrile with silicone diaphragm, (Cole-Parmer, UK) in the middle of long outlet tube (Fig. 1).

### Cell viability

Cell viability was assessed using the Alamar Blue assay (Thermofisher, UK) where the metabolic activity of respiring cells reduces resazurin to resorufin. A 10% v/v solution of Alamar blue in phenol-red-free and FBS-free media was added to the cells (2D or in 3D) and incubated for 4 h at 37 °C. Acellular wells (for 3D culture, compressed collagen scaffolds without cancer cells) were used as controls. Absorbance values were measured using a microplate reader (BMG, Labtech, UK) at a wavelength of 570 nm and 600 nm and the percentage reduction of Alamar Blue reagent was calculated as per the manufacturer’s instructions.

### Gene expression analysis

Expression levels of Ki67, Bcl2, caspase-3, caspase-9, HIF1A, vimentin, snail, MMP14, cadherin-11 and HER2 genes involved in proliferation, apoptosis, hypoxia and EMT was analysed using qRT-PCR. Total RNA was extracted using an RNeasy Micro kit (Qiagen, Germany) according to the manufacturer’s instructions. The quantity and purity of RNA was assessed using a Nanodrop spectrophotometer (Thermofisher Scientific, USA) at 260/280 nm and RNA integrity assessed by agarose gel electrophoresis. Complementary DNA (cDNA) was prepared using the QuantiTect Reverse Transcription Kit (Qiagen, Germany) according to the manufacturer’s instructions. To ensure that no genomic DNA remained in the extracted RNA, sample were prepared omitting the reverse transcriptase from the cDNA production process. Similarly, samples without addition of cDNA were used in the qRT-PCR assay. Primer efficiency (linearity) was assessed prior to undertaking the sample analysis and qRT-PCR reactions prepared using the Quanti-Nova SYBR Green kit (Qiagen, Germany) following the manufacturer’s instructions. A Rotor Gene real-time cycler (BioRad, UK) was used with 40 cycles of the following cycling conditions: denaturation 5 s at 95 °C, combined annealing/extension 10 s at 60 °C. The sequences of the forward and reverse primers for the genes studied in this project were as follows: Bcl2 forward 5′ TCGCCCTGTGGATGACTGA 3′, reverse 5′ CAGAGACAGCCAGGAGAAATCA 3′; caspase-3 forward 5′ CAGCAAACCTCAGGGAAAC 3′, reverse 5′ TCACCCAACCACCCTGGTCTT 3′; caspase-9 forward 5′ GCTCTTCCTTTGTTCATCTCC 3′, Reverse 5′ GCTGCTTGCCTGTTAGTTC 3′; HIF1A forward, 5′ CGTTCCTTCGATCAGTTGTCThe 3′, reverse 5′ TCAGTGGTGGCAGTGGTAGT 3′; vimentin forward, 5′ AGATGGCCCTTGACATTGAG 3′, reverse 5′ CCAGAGGGAGTGAATCCAGA 3′; snail forward, 5′ TTTCTGGTTCTGTGTCCTCTGCCT 3′ reverse, 5′ TGAGTCTGTCAGCCTTTGTCCTGT 3′; MMP14 forward, 5′ ATAAACCCAAAAACCCCACC, reverse 5′ AAACACCCAATGCTTGTCTC 3′; CDH11 forward, 5′ CCCAGTACACGTTGATGCCT 3′, reverse 5′ GACGTTCCCACATTGGACCT 3′; Ki67 forward 5′ CCACACTGTGTCGTCGTTTG 3′, reverse 5′ CCGTGCGCTTATCCATTCA 3′; HER2 forward 5′ AACTGCACCCACTCCTGTGT 3′, reverse 5′ TGATGAGGATCCCAAAGACC 3′. Reference gene: GAPDH forward 5′ GACAGTCAGCCGCATCTTCT 3′, reverse 5′ TTAAAAGCAGCCCTGGTGAC 3′. The expression level of reference gene GAPDH was used as an endogenous control gene for all the samples.

### Protein analysis

Vimentin and HER2 protein levels were assessed using immunostaining and western blotting.

### Immunostaining

Cancer cells grown in 2D were evaluated for levels of vimentin and HER2 proteins as described previously^22,53^. First, tumouroids were washed with PBS and fixed with 10% v/v formaldehyde. They were permeabilised using 1% w/v BSA with 0.2% v/v Triton-100 in PBS at room temperature for 1 h and washed with PBS three times for 5 min prior to immunostaining. Rabbit monoclonal vimentin antibody applied at dilution of 1:100 (#5741, Cell Signalling Technology,) and rabbit monoclonal anti-ErbB 2 antibody (ab134182, Abcam) at dilution of 1:250 in 1% w/v BSA with 0.2% v/v Triton-100 in PBS and incubated at 4 °C overnight followed by 1 h incubation at room temperature and washing in PBS as above. The secondary antibody, goat anti-rabbit IgG, Alexa Fluor 488 (Abcam) was diluted in 1% w/v BSA with 0.2% v/v Triton-100 in PBS at dilution of 1:500 and incubated with the tumouroids for 2.5 h at room temperature and washed in PBS as above. Finally, the nuclei were counterstained using DAPI and cells were imaged using an Apo Tom 0.2 fluorescent microscope (Zeiss, Germany).

### Western blotting

Cancer cells cultured as 2D monolayers were harvested using trypsin–EDTA and the cells pelleted using standard tissue culture techniques. Cells cultured as 3D tumouroids in 24-well plates were pre-treated with 200 units/mL collagenase I (Gibco, UK) in HBSS buffer (450 µL per well) for 60 min on a plate shaker at 37 °C, centrifuged at 1,700×*g* for 5 min and the cell pellet collected. SDS PAGE and western blotting was performed using the cell pellets as described previously 18. The resulting blots were incubated with rabbit monoclonal anti-vimentin antibody (Cell Signalling Technology, USA) at 1:100 and rabbit monoclonal anti-ErbB 2 antibody (ab134182, Abcam, UK) at 1:1,000. Rabbit polyclonal anti-GAPDH antibody (Abcam, UK) was used as loading control on the same blot at 1:5,000 for 90 min. Goat polyclonal anti-rabbit IgG (HRP) pre-adsorbed (Abcam, UK) was used as secondary antibody at 1:5,000 for 60 min. The resulting blot was visualised using enhanced chemiluminescence (ECL) detection system (Thermofisher, USA) by adding a mixture of equal parts of peroxide solution and the luminol/enhancer solution on the blot and incubate it for 5 min. The images were captured using a Biospectrum UVP imaging system following 20 and 60 s exposure times.

### Doxorubicin (DOX) treatment

The sensitivity of cancer cells to DOX was assessed by measuring the cell viability using the Alamar Blue assay. For 3D culture, 5 × 10^4^ cells were seeded in each tumouroid for both cell lines. Cells were allowed to grow for a week, the media was removed and DMEM with 2% v/v FBS was added for 24 h. Cells were then incubated with DOX at 0.1 µM, 0.5 µM, 1 µM, 5 µM and 10 µM diluted in serum-free DMEM. Control wells contained medium alone. For the tumouroids prepared under flow conditions replicate experiments (n = 2 per condition) were prepared either in static cell culture or with IFP ± 5 µM DOX for 48 h. All tumouroids were transferred to 24 well plates and the Alamar blue assay was performed as described above.

### Statistical analysis

Statistical analysis was performed using OriginPro software from OriginLab Corporation, MA, USA. The data was analysed for normality and then evaluated using a paired Student’s t-test. P-value ≤ 0.05 were considered statistically significant.

## Supplementary information

Supplementary Figure Legend.

Supplementary Figure S1.

## Supplementary information

nis available for this paper at 10.1038/s41598-020-68999-9.
