# Supplementary material for: Cancer cells grown in 3D under fluid flow exhibit an aggressive phenotype and reduced responsiveness to the anti-cancer treatment doxorubicin
Source: Sci Rep. 2020 Jul 21;10:12020. doi: 10.1038/s41598-020-68999-9 (PMC7374750; doi:10.1038/s41598-020-68999-9)
Supplement: Supplementary file 1 — Supplementary Figure Legend. [file 41598_2020_68999_MOESM1_ESM.docx]

**Supplementary Data - Figure 1.** Western blotting analysis of vimentin protein of MDA-MB231 cells grown under the conditions as indicated. The protein loading control was either β-actin or GAPDH as indicated.
