# Supplementary figures and images for: Cancer cells grown in 3D under fluid flow exhibit an aggressive phenotype and reduced responsiveness to the anti-cancer treatment doxorubicin
Source: Sci Rep. 2020 Jul 21;10:12020. doi: 10.1038/s41598-020-68999-9 (PMC7374750; doi:10.1038/s41598-020-68999-9)

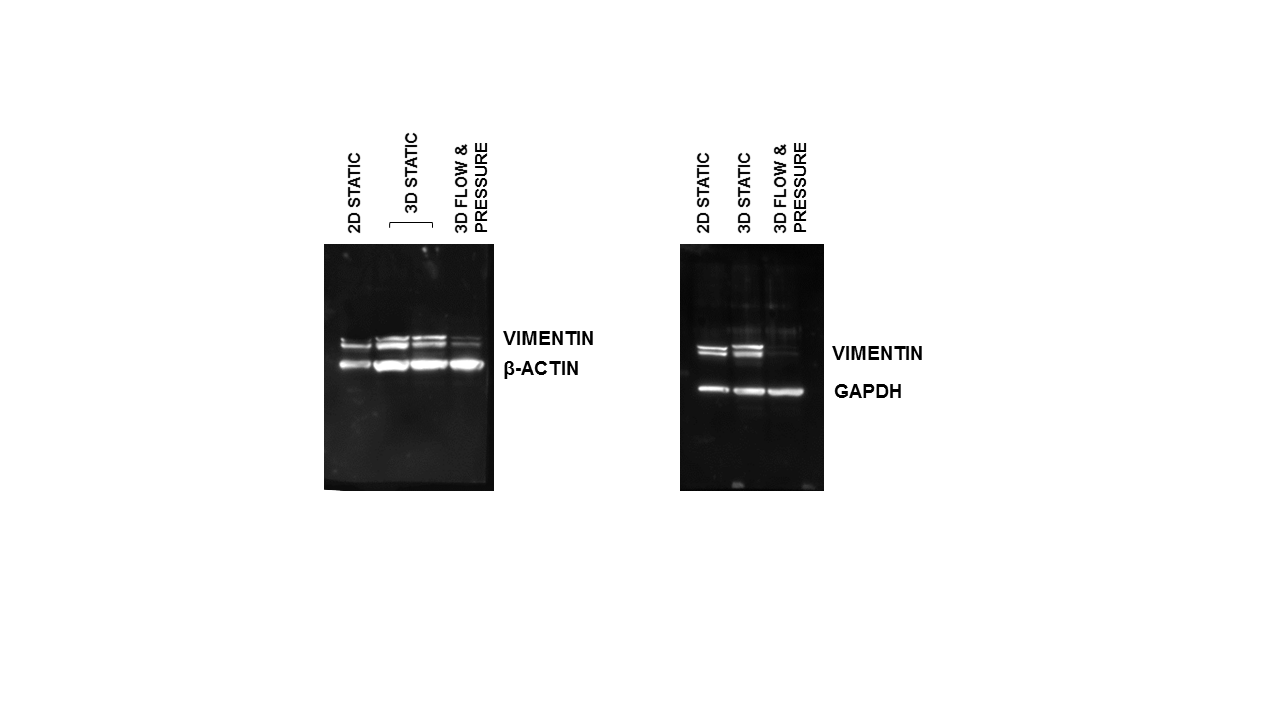

Supplement: Supplementary file 2 — Supplementary Figure S1. [file 41598_2020_68999_MOESM2_ESM.tif]
